# A Lecture Delivered in King's College, London, on Wednesday, 14th March, 1832

**Published:** 1832-07

**Authors:** Gilbert T. Burnett

**Affiliations:** Professor of Botany in the College and to the Medico-Botanical Society, &c. &c. &c.


### BOTANY.

*A Lecture delivered in King's College, London, on Wednesday,* 14*th March,* 1832, *by* GILBERT T. BURNETT, F.L.S., M.B. R.I. R.C.S., Professor of Botany in the College and to the Medico-Botanical Society, &c. &c. &c.

(Continued from page 381 of the last volume.)

ZOOLOGICAL botany is that section of our science which treats of the reciprocal influence of animals and plants upon each other, more especially with reference to their mutual support, defence, migrations, &c. It is, therefore, an extensive topic, as it includes an account of the advantages these two organic reigns severally receive and confer, as well as of the injuries they inflict and sustain, by which the one becomes a natural check to the undue increase and preponderance of the other. This research then opens a very fertile field of philosophical inquiry, and one that I cannot but think has hitherto been much too generally neglected, and yet which, notwithstanding this neglect, is even now far from being barren of facts or devoid of practical interest.

Few persons are aware of the extraordinary influence which even the smaller animals have on plants, and of the important service afforded to the vegetable kingdom, in maintaining a due balance of the various species by the apparent desolation caused by animals; for here the most extensive havoc is often, like the fire of London, a most extensive blessing.

Of these services, insects perhaps afford the most numerous and remarkable illustrations: hence, from the entomological section we shall draw our chief examples.

There is scarcely a plant that is not the peculiar habitat of one or more distinct species, and often the same plant is the domicile of many. The oak is frequented by a variety of other insects besides the numerous species of Cynips already noticed; nor is this a solitary example: other plants are equally infested. Furthermore, the insects themselves, or the diseases they produce, frequently become very important articles of food, medicine, and commerce: for example, the Kermesinus and Coccus, the Lac and Cantharides, the gall-apples of the Salvia pomifera, and the nut-galls of the Levant.

Ample as is the field, few and short are the examples which time will suffer us now to give; and it is to the less pleasing and apparently less profitable, although, on the whole, not less important duties of insects that I wish, in the first place, to direct your attention: for there is truly something quite wonderful in the contemplation of the devastating power of agents that seem so insignificant; something which is, perhaps, more impressive and appalling than when the causes are apparently more equal to the effects.

When pestilence depopulates a land,—when barbarian hordes lay waste the fertile plains of civilised communities,—when savage beasts devour and destroy flocks, herds, and harvests,—when floods, earthquakes, and volcanoes submerge, ingulf, or bury towns, men, and plains,—awful as are the results, still there is something less bewildering, less astounding in the contemplation of such catastrophes, dreadful as they are, because there is a more apparent concordance between the deeds and the agents by which they have been done, than when we consider that creatures so small as locusts can strip, during one visitation, whole forests of their foliage, and destroy every trace of vegetation throughout an extent of several thousand square miles together; and, as was the case when the kingdom of Masanissa thus was scourged, cause upwards of 800,000 persons to die from famine. What are the ravages of beasts,—what the desolation even of earthquakes and volcanoes, when compared to such an unsparing annihilation of men, brutes, and plants by these princes of the powers of the air.

Neither is our astonishment lessened, although its course be turned when we compute their sums, when we find the swarms of these insects to be so vast and dense as to overshadow large tracts of country, and even to intercept the light of day. One of these living clouds, which was three whole days and nights, without apparent intermission, passing over Smyrna, must have been, according to accurate observations made at the time, three hundred yards in depth, upwards of forty miles in width, and nearly five hundred miles in length. Captain Basil Hall calculates "that the lowest number of locusts in this enormous swarm must have exceeded 168,608,563,200,000;" and, "in order to assist the imagination, Captain Beaufort determined that this cloud of locusts, which he saw drifting by when he lay at Smyrna, if formed into a heap, would have exceeded in magnitude more than a thousand and thirty times the largest pyramid of Egypt; or, if they had been placed on the ground close together, they would have encircled the globe with a band a mile and a furlong wide!" Indeed, history tells us that, when these conquering legions are subdued by tempests, their bodies occasionally overspread large tracts of country, even to four feet in depth, and, when driven into the sea, have formed a bank along the shore, three or four feet in height, and extending for fifty miles.

But we need not confine ourselves to foreign illustrations; for we do not lack examples nearer home of somewhat analogous, though far less fatal visitations, as the Aphides and Coccinellæ of our hop plantations, the flies of our turnip fields, and the timber grubs of this and other European countries, will sufficiently declare. The Cossus ligniperda, or great goat moth, is a most powerful and destructive instrument in the hands of nature, and the rapidity with which this power is developed forms one of the not least interesting points of consideration. The larvæ of this insect have been proved by experiment to increase their weight 140 or 200 times in an hour, and, when full grown, to be 72,000 times heavier than when extruded from the egg. The willows near London, especially in the neighbourhood of Hackney, have suffered much lately from the depredations of this insect; but its ravages, and the rapidity of its increase, are nothing in comparison to those of the Termes bellicosus, which lays sixty eggs per minute, and will continue this operation for an almost incredible time, with scarcely any intermission, so that, at this rate, one female might lay 3,600 eggs per hour, or 86,400 in a day; and even a single female of the common flesh-fly, which is not the most prolific of its class, "will give birth to 20,000 young; so that, as my accomplished colleague, the Professor of Geology, observes, there is some ground for the assertions of Linnæus and Wilcke, that three flies of Musca vomitoria could devour a dead horse as quickly as a lion; and that even the smallest insects can commit, when required, more ravages than an elephant, or any of our largest beasts. The importance of these scavengers of nature in removing suddenly effete and useless matter, will be acknowledged on every hand; but although they are employed with much advantage in alternating crops of trees and herbs in forest, grass, and other lands, yet, when they encroach on cultivated grounds, the injury which they commit is lamentable in the extreme.

Several instances immediately in point have been recorded by Mr. Brayley, in his Essay on the Utility of a Knowledge of Nature: he says, "The pine forests of Germany have at various times sustained enormous injury from the attacks of a small beetle, called Bostrichus typographus, 80,000 larvæ having been found in one tree; and, as they feed on the soft inner bark, and multiply thus abundantly, whole forests fall a sacrifice to their voracity, so that, in the Hartz alone, the trees destroyed were calculated at a million and a half; and the inhabitants of this extensive range of country were threatened with a want of fuel to continue their metallurgic operations, and consequently with ruin, entirely dependent as they were upon those branches of the useful arts." Subsequently these Bostrichi, when arrived at their perfect state, in the form of winged beetles, migrated in swarms into Suabia and Franconia, there to commit similar ravages. At length, after repeated injuries, the powers of nature interfered to mitigate the evil, which want of scientific knowledge, as we shall presently shew, had allowed to gain so alarming a head. Between 1784 and 1789, in consequence of a succession of cold and moist seasons, the numbers of this scourge were sensibly diminished: it appeared again, however, in 1790; and, so late as 1796, there was great reason to fear for the few fir-trees that were left.

About twelve years ago, the elm trees in St. James's and Hyde parks suffered much from a similar attack, and whole rows were rapidly being thinned and disappearing, both in the Mall and the Birdcage walk. "As the persons who had the charge of the plantations were entirely ignorant of the true cause of the mischief, and as it was clear that the trees died in consequence of being completely stripped of their bark, rewards were at first offered for the discovery of the delinquents who so mischievously barked them; but these were offered in vain. It was observed, however, (and the observation claims some credit for its ingenuity,) that no more of any tree was barked from the ground than what was easily within the reach of a soldier's bayonet; and this was sufficient to throw suspicion on some unfortunate recruits, of whom more than one was arrested, without producing any diminution of the evil. In vain, too, were persons employed to sit up during whole nights, watching for the enemy; the bark continued to be found every morning at the roots of the trees, and the park-keepers, after all their trouble, could only conclude "that the bark fell off in consequence of something being placed on the trunks in the daytime." About the same time, the elms in the grove at Camberwell, near London, were observed to be undergoing a similar pro-

Very curious cess of destruction; and the proprietors, being equally ignorant of its cause as in the instances just mentioned, the injury was ascribed to the effects of gas escaped from the pipes for lighting the road, which had just been laid down, and legal proceedings were actually commenced for the removal of the nuisance against the gas company which had undertaken the supply." Entomologists, it is true, were aware that the operations of insects were the cause of all this mischief, but unfortunately they were not believed until the disease had reached that pitch which threatened to make remedy hopeless. But at last a naturalist was consulted, and he at once discovered that an insect, called the Hylesinus destructor, had located itself in the parks, and legions of these little fellows were quietly and constantly at work, secretly proceeding in their labours of destruction, in spite and in defiance of Lord Sydney's denunciations. But not only did MAC LEAY discover the cause of this evil: he, in the true spirit of philosophy, likewise directed a remedy to be applied, and these subtle miners became at once obedient to the voice of science, although they had defied the ranger's threats to prosecute them with the utmost severity of the law.

But insects not only do their bidding of destruction; they often defend plants from the aggressions of each other, and frequently protect the weak from the encroachments of the strong. Priority of possession gives many advantages to perennial and hardy over annual and more tender plants; and hence, were it not for the destruction of those by insects, or other animals, they would entirely overrun the land, and stifle the development of these. Hence, when left without interference, as in unreclaimed countries, do we find vast tracts known as the regions of forests, the regions of thistles, and the regions of grass; all of which are more or less intolerant of each other, and maintain for ages their lines of demarcation with the strictest and most arbitrary power: for, notwithstanding the thistle down, as General MILLER states in his Travels through Patagonia, is blown over the bowling-green-like pampas in such abundance that large balls are formed by its association, still few, very few, of the seeds germinate, except in their own peculiar regions. Now, to modify such circumstances, and to restrain the monopolising tendency which all plants exhibit, various animals, and especially insects, are commissioned to curb their tyranny, by means the most simple, and yet the most effectual, that can be possibly conceived. Thus, animals prefer for pasture those situations where their appropriate food is most abundant; and hence they quit those places where little is found, or when they have diminished its abundance. Insects, in like manner, colonise those spots alone, or chiefly, where the fit plants to feed their larvæ grow most freely; and hence it is that the preponderance is restrained; for the destruction or diminution of any over-bearing species will, of course, favor the growth and increase of many that are weaker; and when their mission is performed, that is, when the preponderance is reduced, then the messengers depart, for, as their food is lessened, their numbers are necessarily reduced: and it is one remarkable feature in this extraordinary system of checks and counterchecks, that, unlike man, few of the lower animals are carnivorous: each has its appropriate food, and what will starve or poison some will afford healthy and sufficient sustenance to others. Thus, horses will not touch cruciferous plants, but they will feed on the reed grasses, amidst abundance of which goats have been known to starve; and these latter, again, will eat and grow fat on the water hemlock, which is a rank poison to other cattle; in the like manner, pigs will feed on henbane, while they are destroyed by common pepper; and the horse, which avoids the bland turnip, will grow fat on rhubarb, and take a drachm of arsenic daily with advantage. In the general economy of nature, these idiosyncracies are of extreme importance; for thus each species, even when unsubdued by man, has a natural and wholesome curb placed upon its undue luxuriance, which prevents its increasing to the absolute exclusion of its fellows, and without which the balance could not be sustained. When, for dietetic or other purposes, it is wished that certain plants should occupy exclusively certain extensive tracts, art must then exert her modifying influence, and withhold the hand of nature with unwearied vigilance; for when, as occasionally happens, the artificial bonds are loosened, then do these natural agents demonstrate their power, as, notwithstanding the utmost care, continually is seen.

On the other hand, it not unfrequently happens that animals are of much service in aiding the increase and development of plants, not only by disseminating species, and by many seeds, as the olive and the hawthorn, &c., vegetating more freely after they have passed through their digestive organs; but also by insects aiding in the fertilization of flowers, conveying the pollen of those which are diœcious from the barren to the fruit-bearing organs, and thus becoming the intermediate means of impregnation.

Again, to vary the illustrations, we may note that a small grub, (the larva of the Musca pumilionis,) which its parent deposits in the acrospire of growing corn, does much towards increasing the produce of the plants, which it might have been supposed, from its devouring the plumule, it would irreparably injure. On the contrary, however, the tillering of wheat is much favored by such an attack on the germinating grain, and the number of culms considerably increased. Here it needs not to be told that the abundance of the harvest will greatly depend on the number of culms each grain produces, and these may be, and have been, multiplied to an almost incredible extent. A single grain of barley is recorded to have formed a tuft whence sprang 249 separate stalks, bearing as one season's produce above 18,000 grains; and a single seed of red wheat, which was experimented with by Mr. C. MILLER, of Cambridge, son of the celebrated horticulturist, tillered so freely that it allowed of subdivision into five hundred separate vigorous offsets, which bare 21,109 ears, some of which contained between sixty and seventy grains; and "the wheat, when separated from the straw, weighed forty-seven pounds and seven ounces, and measured three pecks and three quarters, the estimated number of grains being 576,848!" *(Veg. Substan. L.E.K.)*

Of Geological and Mineralogical Botany, I must speak in common; for, although they diverge into different researches, time will not permit me to afford them separate illustrations. The latter is chiefly interesting from the indications it affords of the nature of soils, as by the Patellaria rufa never being known to grow any where excepting on sandstone of different degrees of hardness; the Lichen calcareum on limestone, and so forth; but not only do plants thus indicate the nature of the soils in which they grow, but also, at least as some persons say, (although, as far as I have examined the evidence, I do not think it is sufficient as yet to establish the conclusion:)—some persons (I repeat it) think that not only the nature of the soil, but that of the subsoil also, and even the characters of the subjacent rocks, may be ascertained by a critical examination of the plants growing on the surface, as if nature had written the titles of her works on the outsides of her various volumes. But on this I had rather not at present dwell, as I am far from being satisfied with the instances as yet adduced, curious as confessedly they are; and we have quite enough of wonders that are indubitable to keep us from coveting any that are liable to doubt.

I prefer the term Geological to the older word Fossil Botany, because the former is more comprehensive and includes the latter, which name indeed very imperfectly expresses the extent of this department of our science; which investigates not only the characters of fossil plants, and their distribution in various strata, but also refers to their effects on climate, (for Meteorological Botany is not as yet sufficiently advanced, nor of sufficient extent, to constitute a separate section,) and notes the changes which vegetation is now producing upon the surface of the globe.

The geologist, or natural historian of the earth, must, to do his subject justice, call many collateral sciences to aid him in his researches, and not one of the least important amongst these is botany. To confirm the truth of my assertion, I think that I may appeal with safety to the learned professor of geology in this College; for in his very interesting work he has woven much botanical philosophy; and that part of our united studies which he blends with his, I likewise, although for a somewhat different purpose, include in my researches, and hence we shall be able mutually to assist each other. Thus, the geologist finds in various strata, and in different formations, the fossil remains of numerous plants, and the botanist is indebted to him for the discovery; but the former would reap little comparative advantage from his labour, did not the latter determine whether they are or are not identical with plants now growing on the surface of our globe, and decide the affinity of the extinct remains with the genera and species now existing, and distinguish ever-changing varieties from species, and prove the permanence of specific characters in plants. Often very sorry fragments are all that can be gained of fossil vegetables, and sometimes the impressions of the plants long since decayed are the only memorials which descend to us; and yet these are generally sufficient to shew whether they were similar or dissimilar to such as now are known, and whether the antediluvian flora had plants identical with or equivalent to our own. From relics such as these before us, the vegetable physiologist can often tell what must have been the probable temperature and other physical conditions of the countries where such fossil witnesses are found, in eras so remote that, to the ignorant and the unlearned, they seem impossible to be submitted to the cognizance of existing man. Thus, when plants which we recognise as arboreous ferns and palms, and gigantic reeds, are found in various northern regions,—when plants similar to those which now are known to require intertropical heat are discovered in the temperate and towards the frigid zones, such discoveries attest to us not only the great climatorial changes which the earth has suffered, but they discover also those vast mutations which the apparently everlasting hills have undergone: and thus they register the mutability of what seems most immutable to man. Yes; these dumb mouths indeed do tell a tale of wonder, and in terms that cannot be gainsaid: they tell us that the heights of many of our mountain ranges were once beneath the sea; that the soil of our most fertile and most extensive plains is composed of the fragments of things which, like us and our modern animals and plants, once lived and moved, and had their transitory being upon this earth, although long since they have returned to it again; that the exuviæ of myriads, that the forests of ages, must have been concerned in the production of our bogs and peats, and especially in our coal formations; and furthermore they hint to us that, as they did not, neither do we, live wholly for ourselves, but that, as they conduce greatly to our comfort, so that, after we have lived our little here, our bodies, and those of our contemporaneous plants, like theirs, may be destined to subserve similar important services to generations which shall yet be born, in countless ages yet to come.

Oh, gentlemen, believe me, the feelings with which a botanical philosopher contemplates the various productions of the vegetable world are very different from those with which they are viewed by one unblessed by the light of science! How different is the barren knowledge of the existence of all these things around us, which every one knows to be, from a knowledge of the laws by which they are regulated and sustained. Never, indeed, to my mind, does true wisdom more fully vindicate her majesty and power, than when, as in this case, she thus unfolds a leaf turned down by nature, and reveals to us a record of those changes which long since have been forgotten, (if, indeed, to man they were ever known,) than when she thus turns back the pages of past time, and reads in these majestic tablets of the Creator the history of his wondrous works, as published in the volume of creation. The whole earth, like Ezekiel's scroll, is written over, both within and without: to the ignorant and the thoughtless it may, perhaps, appear to be inscribed with mourning, lamentation, and woe; but to the philosopher it tells a constant tale of miracle and mercy, as HUNTER has well observed on a somewhat similar occasion: "appearances of this sublime nature may be compared to the handwriting upon the wall, which, although seen by many, was understood by few: they seem to constitute a kind of harmonious intercourse between God and man; they are, indeed, the silent language of the Deity."

*Systematic Botany, or Vegetable Diagnosis, the second grand section of our science, objectively considered, includes, when liberally viewed, 1, Phytography or Descriptive Botany; 2, Phytaxonomy, or the Laws of System, as affecting the arrangement of vegetables; and 3, Phytochronology, or the Annals of Botany, comprehending the history of plants both natural and traditional, with the general literature and all other records of the science.

To Phytography, or Descriptive Botany, belongs the study, both theoretical and practical, of the verbal, pictorial, and actual representations of plants, either separately or in union: i. e. it consists in a knowledge of the terms invented to denominate, the figures designed to pourtray, and the means employed to preserve the various individuals of the vegetable world, and their various parts, under all their varied modifications.

The first of these subdivisions has been termed *Glossology,* or the Language of Botany; the second might be named *Botanography,* or the scientific Delineation of Plants; and the third is but a part of the conservator's province, which he designates *Taxidermy.*

The language of botany, called by the older writers *Terminology,* or the doctrine of technical terms, and by De Candolle, who objects to this word, *Glossology,* is a very fundamental part of systematic botany; for, notwithstanding it has been asked, and in a tone that almost precludes reply, "What's in a name?" still we must contend that, in matters of science, nomenclature is a subject of no mean importance.

Botanists have laboured more abundantly, and perhaps more successfully than most other naturalists, to define with precision the terms they use, and to reduce their language to an acknowledged scale. Hence botany has long been famed for its "copia verborum:" and hence arises the brevity and perspicuity of botanical definitions; hence also the succinctness and clearness with which plants may be described, and the few words in which details of the most complex structures may be couched. Instead, therefore, of any exceptions being on this point admissible, it is to be regretted that the glossology of the other natural sciences is less advanced and far less perfected than, with all its faults,

* This section, and two or three other paragraphs, were, on account of time, omitted in the delivery of the first, and introduced into the second lecture; but, to preserve the connexion of the argument, it has been thought better to let them appear in print in the places they were originally designed to occupy.

and we confess them to be many, the language of botany indisputably is, and the only matter for sincere regret is that philosophers cannot universally agree as to the meanings they severally attach to certain terms, nor even as to the terms by which they denote the same or dissimilar ideas; for not only are different words frequently used to express the same meaning, but, what is of far more injurious tendency, to one word very often several different significations are attached. Such indeterminate phraseology is the bane of science, and it were almost to be wished that some philosophic autocrat could compel, by an imperial *ukasse*, the invariable usage of different words for the expression of different ideas, and of similar terms to represent the same.

To prevent the baleful effects of this laxity of language, whenever and wherever it prevails, forms the most anxious part of the professor's and the most irksome of the pupil's duties; for, while current terms are used in science only to express their legitimate ideas, still the collateral significations cannot, and in good truth should not, be entirely overlooked: if we wish to understand those works in which the same words are less properly applied, either through the haste and carelessness of unscholastic writing, or from the errors which an imperfect acquaintance with vegetable physiology has introduced; and which, as our knowledge is day by day increasing, so it is daily rendering the views under which many names were given obsolete, and the dogmas they inculcate incorrect. This last is a difficulty inseparable from a progressive, a rapidly improving state of science; and it is a difficulty we need never wish to be removed. It is one that richly rewards us for any trouble it occasions, and its difficulties are readily overcome by all who are willing to learn *facts* as they exist in nature, without any exclusive deference to *words;* for, when things themselves are understood, the language in which they are clothed, although important, is of secondary importance, and though different in different ages, and differently used by different persons, can scarcely obscure the subject greatly.

I always have been, and still purpose to continue, very jealous of the introduction of new names, well knowing that a workman may become burdened, and his labour hindered, by the multiplicity and complexity of his instruments and apparatus: yet I do not mean by this to avow any slavish adhesion to terms which are manifestly incorrect, or any fear of introducing a new word when a new idea requires representation, or an old one is either to be viewed in a new light, or can by a new term be more decidedly expressed; and hence I shall occasionally lie under the necessity of employing some technicalities that you will not meet with elsewhere; but these will never be introduced without a full explanation of their meanings, and in doing this I shall follow the example of the older botanists, and employ words as far as possible significant of the ideas intended to be conveyed, and with the same significations, whenever it can be done, in which they are ordinarily used.

One common word, however hypercritical the change may sound, I will confess that I do wish to see a little altered; and as it is one of the first we shall be compelled to use, and one that, of all, will be the most frequently employed, perhaps it will be as well at once to state it, and thus early to anticipate any objection that may not impossibly be raised to the substitution of the word *vegetal* for vegetable, in the drafts and diagrams which have been drawn out to assist in the explication of these lectures. This has been done designedly, and not by chance; for, as I have elsewhere observed, "How such an irregular and inharmonious word as veget*able* became established in our tongue, to the prejudice of the legitimate and more elegant (although now regarded as dis-*able*-d) vegetal, can scarcely be conjectured. This latter word, long all but obsolete, still has good authority to boast: Butler writes, "as from a seed, all sorts of *vegetals* proceed;" Burton also prefers this form; he says, "The earth yields nourishment to *vegetals,* sensible creatures feed on *vegetals,*" &c. &c. Both *végétal* and *végétable* are current words among the French, and, as the latter can scarcely be exploded now, it is to be hoped that both will soon become equally familiar terms with us. My acute and learned friend, Mr. George Field, in his "Analogy of the Physical Sciences," has also well observed, "It is to be desired that custom should authorize the substitution of *vegetal* for vegetable, whether used as a substantive or adjective; for if the terms animal and mineral be more proper than anim*able* and miner*able,* then, by correct analogy, *vegetal* is more proper than veget*able.*" If custom, however, refuse to admit the euphonious vegetal as copartner with animal and mineral, at least let consistency pervade the whole, for *animable* and *minerable* are alone fit company for the cacophonious *vegetable.* Look on this column, and on that.

| | |
|---|---|
| Animal | Animable |
| Vegetal | Vegetable |
| Mineral | Minerable. |

"Utrum horum mavis accipe."

Notwithstanding the acknowledged advantages thence derived, the language of botany has often been regarded with fear, and we still find it to be that part of the study most commonly objected to. This is not the only case, however, in which the facilities afforded by science have been ignorantly mistaken for difficulties inseparable therefrom: other instances could be given in which what we might perhaps be allowed to call the almost too exclusive privileges of botany, have been described as its peculiar disadvantages by those who little understood their import, and consequently were led to underrate and misrepresent their value. To these I shall not further now allude, but confine my present animadversions to this outcry against *hard words*, as the technicalities of science have foolishly been called; whereas, it should be remembered, as JOHNSON says, that "words are only hard to those who do not understand them;" and, so far from our terms being really hard, the language of botany is more easy and intelligible, because it is more copious and precise, than that of the other natural sciences. To take an illustration: an exclaimer against hard words would, perhaps, when describing the figure of this ball, be content to say that it is round; another would declare this ring to be also round; and a third would as confidently assert both a cylinder and a coin to be round likewise; yet how various are these figures, and how greatly do they differ in their rotundity. Such wavering and inconstant language may suffice to express the ordinary ideas of ordinary minds, and nothing more ; it is fit for such persons and purposes, and for such alone; and botanists may be therefore well content to bear the reproach even of pedantry for devoting distinct words to the representation of such distinct and varied figures, and for always, as in this case, preferring philosophic perspicuity to popular prejudice, and for insisting upon the use of different words for the expression of different ideas. Botanists are not of that sect which declares that "language was given to man to conceal his thoughts:" we rather side with those who teach that the true purpose of language, and the great art of speaking, is so to express your ideas that they not only may, but must be understood, so to speak that your hearers not only may understand, but so that they shall not be able, by any possibility, to misunderstand your meaning; and the nearer any language approaches to this state, the nearer it approaches to perfection. Hence, although such persons as confine their researches to the books of men, without reference to the book of nature, who study names, not things, may bewilder themselves amidst the mazes of unintelligible technicalities which then, indeed, become uncouth and barbarous terms, which, in the hands of the skilful, are instruments of power: still, when botany is made, as it ought to be, a study of things, not a study of names, I will venture to affirm that there is nothing in its language that need excite our fear; for there is no science more easy in the acquirement, none which so soon rewards the pupil for a small, a very small, outlay of study and attention.

I would, therefore, recommend all persons to indulge themselves in the delights of botany; for they will find it a relaxation rather than a toil, an amusement rather than a labour; a profitable pastime in youth, an agreeable occupation in manhood, and a gratifying research in honourable old age; when having, as we hope all who are educated here will do, passed through this world useful to their generation, and not useless to themselves; when having acquired, by meritorious exertion, a competency of wealth and a sufficiency of fame, they may retire, like Cincinnatus, from the senate to the field, and in a garden—(what pleasure is there not associated with the very name of a garden, it bespeaks at once serenity and ease,)—in a garden forget awhile this world, its turmoils and cares, before they are summoned to quit it for a better.

(To be concluded in our next.) p. 111.

---

